# Spatial repellents and malaria transmission in an endemic area of Cambodia with high mosquito net usage

**Published:** 2017-08-01

**Authors:** Jacques D. Charlwood, Tom Hall, Somalay Nenhep, Emily Rippon, Ana Branca-Lopes, Keith Steen, Bruno Arca, Chris Drakeley

**Affiliations:** 1 Department of Entomology, Liverpool School of Tropical Medicine, Liverpool L3 5QA, UK; 2 SOREMA, IRD, Phnom Penh, Cambodia; 3 London School of Hygiene and Tropical Medicine, Kepple Street/Gower Street, London WC1E, UK; 4 Centro Nacional de Malaria, (CNM) Phnom Penh, Cambodia; 5 Department of Public Health and Infectious Diseases, Division of Parasitology, Sapienza University, Piazzale Aldo Moro 5, 00185 Rome, Italy

## Abstract

**Background:**

The spread of artemisinin resistant malaria from SE Asia to the rest of the world remains a threat that will only be ended by eliminating malaria from the region. Novel control approaches are required to mitigate this threat. Spatial repellents (SR) are one such approach. We therefore conducted a multiple cross-over experiment from April 2013 – April 2014, in which all houses in one of two villages in Mondolkiri Province, Cambodia were alternately supplied with an emanator of the spatial repellent metofluthrin per 30 m^3^ of protected area to cover all potential peridomestic areas where people might spend their time before sleeping. Emanators were replaced every month for a three-month period.

**Material and methods:**

Mosquito densities were simultaneously monitored in each village for two weeks every month using six CDC light-traps/night run from 18.00 to 07.00 hrs inside bedrooms and malaria prevalence, seroconversion and gSG6 protein rates assessed from prevalence surveys. After emanators were installed in the first village they were installed in the second village for a further three-month period and following that were again used in the initial village for a further three months. Surveys were undertaken before the initial installation of the emanators and at each cross-over point.

**Results:**

*Anopheles dirus* densities were highest in houses closest to the forest. Transmission rates were low even before the application of the emanators. Perhaps due to the low levels of malaria transmission in Mondolkiri no significant relationships were found in *Plasmodium* cases or seroconversion rates between villages, surveys or by intervention. Adult males, who might spend more time unprotected in the forest at night, appeared to be at greater risk of becoming infected with *P. falciparum* malaria as compared to women or young children.

**Conclusion:**

At the malaria transmission levels present in Mondolkiri the metofluthrin emanators evaluated had no observable effect on malaria prevalence. This may be due to confounding by low prevalence rates.

## 1 Introduction

Treatment failure of artemisinin combination therapies (TFACT) against malaria parasites in SE Asia, particularly Cambodia, is potentially a major problem for malaria control worldwide [1-3]. Protection may be provided by mosquito nets if available and used correctly, however, many people in Cambodia, even in relatively isolated villages, participate in practices that may expose them to vectors before going to bed. Alternative strategies for early-evening and/or outdoor protection are, therefore, required. Topical repellents which have been shown to reduce transmission when used in combination with Long Lasting Insecticide Treated Nets (LLIN) are one obvious possibility [4] although they do involve daily application and people may not use them even if available [5]. Spatial repellents (SR), which release volatile chemicals to prevent biting in a volume of air, are another alternative product category [6-9]. A trial in Indonesia demonstrated protective efficacy against new malaria infections using metofluthrin coils [9] and Hill *et al.* [10] demonstrated a synergistic effect of LLINs with transfluthrin coils against malaria in China. The development of formulations with high vapour action at ambient temperatures has led to the elaboration of devices that work without requiring the application of heat [11]. Such devices have the additional advantage that they will work for an extended period of time (weeks instead of hours), which makes them an attractive alternative for control of all disease vectors. Studies in different contexts are, however, needed to demonstrate the public health value of such spatial repellents in contexts of low, or residual, transmission [12].

Although not designed as a malaria control tool (due to their high cost) slow-release emanators made of dual layer polyethylene mesh, impregnated with 10% (w/w) metofluthrin, have recently been shown to reduce landing rates of outdoor biting of secondary vectors of malaria in Cambodia [13]. When used at an individual level they reduced landing rates by 48% but when applied on a wide scale it is possible that an area-wide effect could be obtained, such as is observed with mosquito nets or topical repellents [14,15].

Previous studies in Cambodia took place in areas where there was little or no malaria transmission and *Anopheles dirus,* the principal malaria vector in the region, was almost absent [13]. Thus, the effect of usage on *An. dirus* mediated transmission could not be rigorously investigated. The effect of a metofluthrin spatial repellent emanator on malaria prevalence when used indoors and in peridomicilliary settings, was, therefore, examined in a cross-over study design in two isolated villages of Mondolkiri Province where autochthonous, low level, malaria transmission occurs.

## 2 Materials and methods

### 2.1 Description of the study site

Two villages, Ou Chra and Pu Cha, located in an area of diminishing forest in Mondolkiri Province, Cambodia, were chosen for the study. These villages, which have been previously described [16,17], are in one of the remaining areas of Cambodia that continues to have endemic malaria. The great majority of residents in both villages are ethnic Phnong, an aboriginal group mainly found in Mondolkiri that rely on subsistence agriculture. Long-lasting insecticide treated nets (LLINs) were distributed free of charge by the national malaria programme in 2006 and their continuing availability represents the main vector control method in the province [18]. Both Ou Chra and Pu Cha had a Village Malaria Worker (VMW) trained to diagnose suspected malaria cases by using a rapid diagnostic test (RDT), administer artemisinin-based combination therapy (ACT), and refer patients to the nearest public health facility [19].

At the start of the study all 34 houses in Ou Chra and the 51 houses that formed the closest hamlet of the village of Pu Cha were mapped with hand held GPS units (Supplementary file 1) and consent to participate in the study was obtained. Houses were photographed and a to-scale plan (to the nearest 10 cm) of houses drawn. The height of the roof from the floor was also determined so that the volume of air inside the different rooms could be calculated. Residents were asked about their evening activities, their use of nets and, in order to determine their relative socio-economic status, their ownership of certain goods (data not shown here). Baseline collections of mosquitoes were undertaken for six months and, at the end of this time, an initial parasitological survey was undertaken to determine malaria prevalence in the two villages. After this, a cross-over experiment in which each village acted alternately as a control, for three months, or as the intervention village. Houses in the intervention village were provided with metofluthrin emanators, supplied by Sumitomo Chemical Co. Ltd. (Hyogo, Japan).

The emanators were made of polyethylene dual layer (15 x 8 cm wide) 3–4 mesh held in an open plastic frame impregnated with 10% (w/w) metofluthrin (2,3,5,6-tetrafluoro-4-(methoxymethyl)benzyl(*EZ*)-(1*RS*)-cis-trans-2,2-dimethyl -3-prop-1-enylcyclopropanecarboxylate). According to manufacturers specification a single emanator can protect 30 m^3^ of space for 4 weeks. The total estimated volume of each area requiring protection (which included verandas in front of television sets) was determined according to where people stayed in the evening and to the available openings through which mosquitoes might enter. The number of emanators required to meet specifications along with their optimum location (such as by the eave gap, if there was one, or above the television) was established. Wires or nails were put into place such that the emanator was in a location where it was potentially exposed to air movement. Locations were chosen on an *ad hoc* basis according to the experience of the investigators. Thus, if a room required five emanators to be fully covered the five locations used for installation were chosen to be sufficiently separated so that coverage would be complete and that locations with the largest opening were covered. They were installed above head height (circa 2.4 m) so that they did not interfere with other activities and were out of reach of children. The initial installation of the wire or nails was done before the application of the emanators. The emanator came with a plastic clip so that, once the initial wire or nails had been prepared, installation and replacement in a house was a few seconds’ work and installation (and replacement) in all houses in the intervention village could be completed in a few hours. Emanators were replaced every 4 weeks. Unfortunately, placebo products were not available so that the experiment was not blinded between villagers or project staff.

Following the baseline entomological and parasitological surveys emanators were installed in all houses in Ou Chra in June 2013 and replaced at monthly intervals for three months. The intervention was then crossed over into Pu Cha and Ou Chra became the control. A total of three crosses occurred: September 2013, December 2013 and March 2014. Simultaneous entomological and parasitological surveys were conducted during the three-month period for each cross.

### 2.2 Entomology

Entomological assessment was designed to estimate exposure to potential malaria vectors inside houses. CDC light-traps hung 1.5 m off the floor at the foot of the bed in which sleepers were protected by an LLIN were used to sample potential malaria vectors indoors. For 2 weeks each month, from April 2013 to March 2014, light-traps were simultaneously run from 18.00 to 06.00 hrs the following morning in each village in a random set of houses such that by the end of the two weeks all 34 houses in Ou Chra and a random set of houses from Pu Cha had been sampled. In addition, almost daily collections were undertaken during 12 of the 21 weeks from 3^rd^ of May to 19^th^ of June 2013 (ISO week 18 to 25) in Ou Chra and during seven weeks in Pu Cha. Sentinel collections were also undertaken at the edge of what remained of the forest in Ou Chra. Collected mosquitoes were dissected to determine parous rates and the duration of the gonotrophic cycle, the results of which have been reported elsewhere [17]. In order to obtain a broader picture of the mosquito fauna a small number of light-trap samples were run over a pig sty close to one of the high density houses adjacent to the forest and a number of collections using a ‘mosquito magnet’ MMX trap [20] were run in two locations close to the forest. The mosquitoes from these collections were not included in the overall analysis.

A sample of the mosquitoes morphologically identified as *An. dirus* s.l. were identified to species using the PCR-protocol as described by Walton *et al.* [21]. Unfortunately, project resources did not allow for the identification by PCR of the remaining species or samples.

### 2.3 Parasitology

During each survey, over a period of 3 days in Pu Cha and 2 days and a morning in Ou Chra, all residents were invited to attend a parasitology investigation under the house of the village leader. When attending the survey, a persons’ temperature was taken; they were asked at what time they had gone to bed the previous night, if they owned a mosquito net and if they had used it the previous night. People attending the surveys with a fever (a temperature ≥37.5°C) were tested with a Rapid Diagnostic Test (RDT) (OptiMal®; TCS Biosciences, Buckingham, UK) for the presence of *Plasmodium* antibodies and, if positive, were given first line malaria treatment as recommended by the National Malaria Control Programme of Cambodia.

Filter paper samples for subsequent PCR determination of parasite prevalence were obtained from finger prick blood. Blood was also eluted into 150 μl Eppendorf tubes containing EDTA that facilitated the separation of serum from the clot. All blood and serum samples were kept on ice and transported to Phnom Penh for storage at -20°C until shipment to the United Kingdom for processing. Serum samples were tested for antimalarial IgG antibodies against merozoite surface protein-1_19_ (MSP1_19_) and apical membrane antigen-1 (AMA1) using an indirect ELISA.

Samples from each dried blood spot were removed with a standard hole punch (providing a sample equivalent to 1 μl in a 10 μl reaction) and DNAs were extracted using a previously described chelex/boiling method [22]. A high-throughput assay based on real-time PCR using TaqMan probes and real-time Nucleic Acid Sequence-Based Amplification (NASBA) as described previously [23-25] was used to detect the presence of malaria parasites in the blood spots.

Collected sera were tested for antimalarial IgG antibodies against merozoite surface protein-1_19_ (MSP1_19_) and apical membrane antigen-1 (AMA1) using an indirect ELISA at 1/1000 and 1/2000 dilutions respectively.

High absorbance ELISA plates (Immulon 4 HX) were coated with 50 μl of MSP1_19_ or AMA1 at 0.1795 μg/ml and 0.5 μg/ml respectively and kept overnight at 4°C. Plates were washed using PBS plus 0.05% Tween 20 (PBS/T) and incubated for 3 hrs at room temperature (RT) with 150 μl of 1% (w/v) skimmed milk powder (Marvel, UK) in PBS/T. Plates were washed again and 50μl of samples were added in duplicate to each plate. A pool of sera from Tanzania, a highly endemic area in Africa, was titrated on each plate as a positive control. Following another overnight incubation at 4°C the plates were washed (PBS/T) and incubated (3 hrs, RT) with 50 μl of horseradish peroxidase-conjugated rabbit anti-human IgG (Dako, 1/5000 in PBS/T). All plates were developed using 100 μl of OPD substrate solution (15 min, RT in the dark) and reactions were stopped with 25 μl of 2 M H_2_SO4. Plates were read immediately at 492 nm and optical density (OD) values recorded.

The prevalence of gSG6 protein, found in the saliva of *Anopheles* can be used as a marker of exposure to biting females [26-28]. It appears to be specific to *Anopheles* and because of its synthetic nature reproducibility of the immunological assay is to be expected [26-28]. In Africa, it elicits an antibody response that is correlated with the level of exposure to *An. gambiae* bites [29]. For the Elisa immulon 4 HX plates were coated overnight at 4°C with 50 μl of gSG6 (5 μg/ml). After washing, with PBS/T, wells were blocked (3 hrs, RT) with 150 μl of 1% w/v skimmed dry milk (Marvel, UK) in PBS/T, washed again and incubated overnight at 4°C with 50 μl of serum (1:200) in blocking buffer. In order to allow for standardization of OD values between day-to-day and inter-plate variation sera were analysed in duplicate with antigen and once without antigen (coating buffer only) and positive control sera (1:200 in PBST/Marvel). Positive controls were obtained from staff from the Liverpool School of Tropical Medicine who had been fed on by a colony of *An. gambiae.* Negative control sera were obtained from staff of the London School of Hygiene and Tropical Medicine who had not been exposed to *An. gambiae* bites.

Following another overnight incubation at 4°C plates were washed and incubated (3 hrs, RT) with 100 μl of polyclonal rabbit anti-human IgG/HRP antibody (Dako, 1:5000 in blocking buffer). After washing colorimetric development was carried out (15 min, RT in the dark) with 100 μl of OPD. The reaction was terminated by adding 25 μl of 2M H_2_SO_4_ and the OD_492_ determined using a microplate reader. Results were processed as previously described [30]. Positive control sera were analysed to allow standardisation of OD values and negative control sera, taken from individuals with no recent travel to malaria-endemic countries, were used to calculate IgG seroprevalence. A cut off for seropositivity of samples was determined as the mean OD of unexposed sera plus 3 standard deviations.

### 2.4 Data analysis

IgG levels were expressed as final OD calculated as the mean OD value with antigen minus the OD value without antigen. The OD values for the malaria antigen were normalised using the standard curve on each plate and the results dichotomised into positive or negative using a mixture model to determine a cut off, as described previously [31]. Cut offs for PfAMA were 0.166 norm OD and for PfMSP were 0.249 norm OD.

A reversible catalytic conversion model was used to fit age seroprevalence data and estimate seroconversion rates (SCR) [32]. SCR can be used to compare sites and the effect of interventions. The model was used to generate age seroprevalence curves, from which a SCR representing the force of infection was calculated. Profile likelihood plots were used to test for a step change in SCR, as previously described [32] and, if present, identify the most likely age at which this occurred [31]. Models with two SCRs instead of one were assumed if the likelihood ratio comparing models indicated a statistically significant change (p < 0.05).

### 2.5 Ethics

The study was approved by the National Ethics Committee for Health Research in Phnom Penh, Cambodia (0032/ NECHR, dated 4 March 2013). Informed consent was obtained for all respondents.

## 3 Results

### 3.1 Population parameters

A total of 145 people were registered in Ou Chra and 309 in Pu Cha. The age and sex distribution was similar between the two villages (Table 1), half being females in each village and 45% and 41%, respectively, being under 15 years of age. The ownership of various household goods was also similar between villages.

**Table 1:** Age structure, by sex, of the inhabitants of the two study villages.

	Ou Chra		Pu Cha	
Age group	Female	Male	Female	Male
<1	1	4	4	2
1-4	3	7	14	20
5-9	20	8	22	30
10-14	9	13	21	16
15-19	8	9	21	19
20-35	20	14	39	30
Over 35	13	16	34	37
Total	74	71	155	154

### 3.2 Metofluthrin

A total of 86 emanators (mean of 2.5 emanators per house) were installed in Ou Chra each month when it was the intervention village and 125 (mean of 2.1 per house) in Pu Cha. Two of the houses in Ou Chra, were cook houses and people did not sleep in them. Emanators were, nevertheless, installed since that was where the families ate their evening meal. No evaluation of the products integrity was undertaken during the study although caged specimens of *An. dirus* from a colony maintained in Phnom Penh were knocked down within 15 minutes when exposed at 30cm from a random set of four similar samples in *ad hoc* bioassays.

### 3.3 Entomology

**Figure 1:**
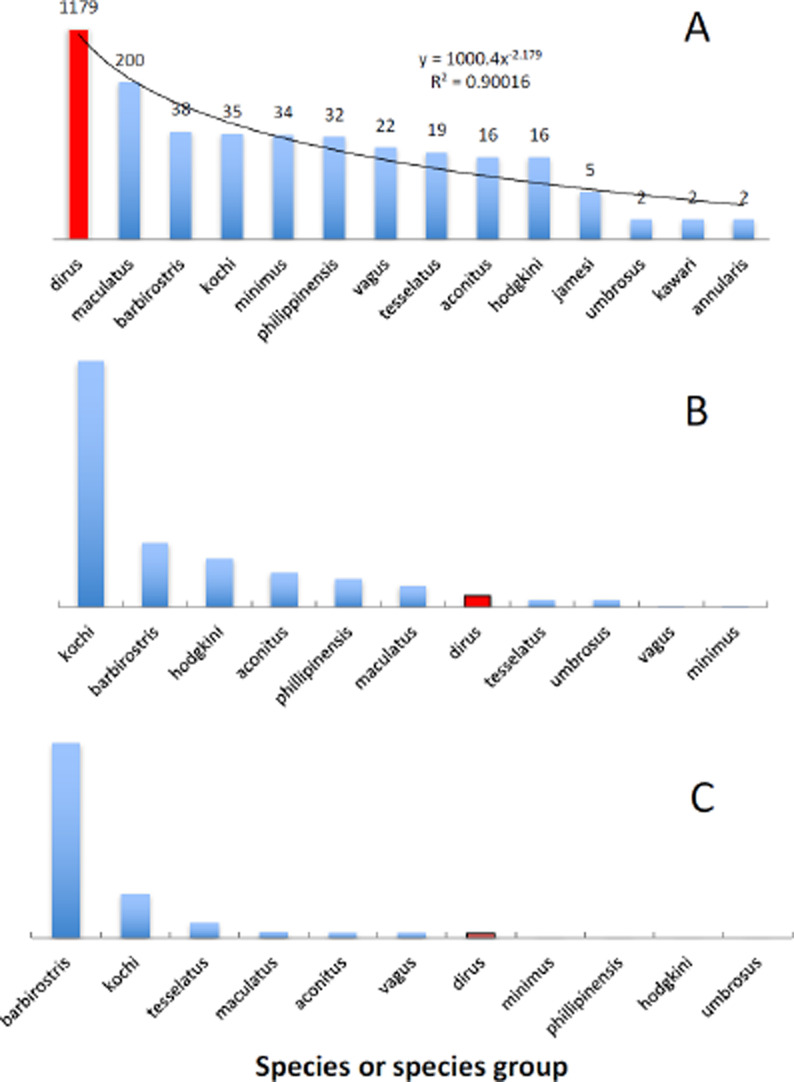
Species of anophelines collected from Ou Chra in rank order (on a log scale). A – CDC light-traps inside houses, B– CDC light-traps over a pigsty, C– from an MMX trap. Note the preponderance of *An. dirus* (in red) inside houses compared to the other trap types.

**Figure 2:**
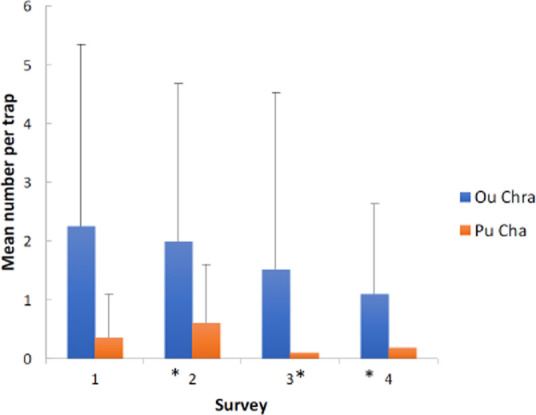
Mean numbers of *An. dirus* collected in CDC light-traps indoors per three-month period by village when metofluthrin emanators were or were not present in the village. (The asterisk indicates that metofluthrin was present in the village).

All 102 *An. dirus* s.l. identified to species by PCR were *An. dirus s.s.*. We therefore presume that this was the only member of the complex present in the study area. Altogether 554 light traps were run in Ou Chra (a mean of 14.6 trap nights per house, excluding the 86 from the sentinel houses) and 406 in Pu Cha (a mean of 6.9 trap nights per house). Fourteen species or species complexes of anophelines were identified from light-trap collections in Ou Chra and nine from Pu Cha. *Anopheles dirus* was the most common anopheline caught in light-traps in Ou Chra and the second most common species in Pu Cha (where *An. maculatus s.l.* was the most common species) (Figure 1). In light-traps run close to a pigsty and in the MMX trap in Ou Chra *An. dirus* was only the seventh most common species collected (Figure 1). Even excluding the high-density sentinel houses close to the forest in Ou Chra the mean number of 1.85 (s.d. 2.2) *An. dirus* per trap was more than six times the 0.275 (s.d. 0.46) per trap recorded from Pu Cha. When the houses closest to the forest were included the mean number collected in Ou Chra was 2.52 (s.d. 3.29), a nine-fold difference. Figure 2 gives the mean number of *An. dirus* (with 95% confidence intervals) from light trap collections in the two villages.

As rainfall increased at the start of the wet season (from week 10 to week 25) so did the number of *An. dirus* (correlation between numbers and rainfall= 0.852). Numbers of *An. dirus* collected decreased only slightly with increasing house height. Mean numbers of *An. dirus* in Ou Chra decreased in each three-month period independent of the presence of metofluthrin. Numbers in Pu Cha, however, were lower when the metofluthrin was present but this difference was not significant (Figure 2).

### 3.4 Parasitology

A total of 1180 samples were collected in the four surveys (661 females and 519 males) (data not shown here). Parasitological results are given in Table 2. Prevalence was always very low even before the application of the intervention. Overall, nine cases of *P. falciparum* (3 females and 6 males) were recorded from Ou Chra and ten from Pu Cha (4 females and 6 males) whilst nine cases of *P. vivax* were recorded from Ou Chra (3 females and 6 males) and 10 from Pu Cha (4 females, 5 males and one sample where the sex was not recorded) (Table 2). Three of the 12 *P. vivax* positive attendees were positive in two consecutive surveys, one attendee had a mixed infection and one, a seventeen-year old male, was positive for *P. falciparum* in June and *P. vivax* in April (but he only attended these two surveys). All but three of the 17 positive *P. falciparum* attendees were over 17 years of age whilst 7 of the 12 people with *P. vivax* were under 15 years of age.

**Table 2:** Parasitological results, by village, sex and metofluthrin presence, determined by PCR from the four surveys.

Survey month	Metofluthrin		Total tested		*P. falciparum* positive				*P. vivax* positive			
	Ou Chra	Pu Cha	Ou Chra	Pu Cha	Ou Chra		Pu Cha		Ou Chra		Pu Cha	
					Male	Female	Male	Female	Male	Female	Male	Female
Jun	No	No	114	237	2	2	1	1	2	2	1	1
Sep	Yes	No	100	243	3	0	1	1	3	0	1	1
Dec	No	Yes	89	200	1	1	3	2	1	1	3	2
Apr	Yes	No	55	112	0	0	0	0	0	0	1*	

* one positive sample in which the sex was not recorded

Given the low prevalence rates it is not surprising that according to the results of parametric tests there were no significant differences in *Plasmodium falciparum* or *P. vivax*/*P. ovale* distribution between villages or between surveys. Using the data from all the surveys combined no differences in the distribution of *Plasmodium* cases by age group were identified.

### 3.5 Serology

Optical density (OD) values were generally low, as is expected in a region of relatively low transmission intensity. ODs followed the usual trend of increasing with age. This was more pronounced for *P. falciparum* than *P. vivax* antigens.

**Figure 3:**
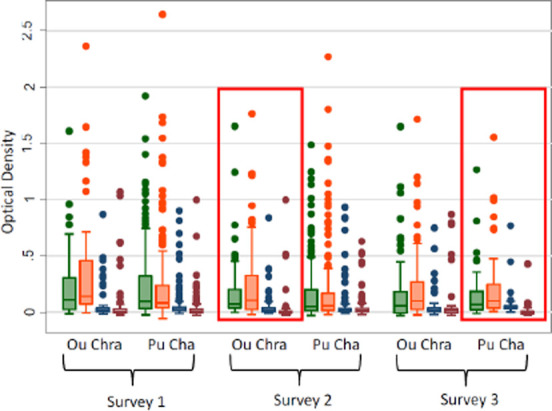
Box plot describing the range of normalised optical densities of antibody responses to PfAMA-1 (green), PfMSP-119 (orange), PvAMA-1 (blue) and PvMSP-119 (red). Red boxes denote the application of the intervention.

**Figure 4:**
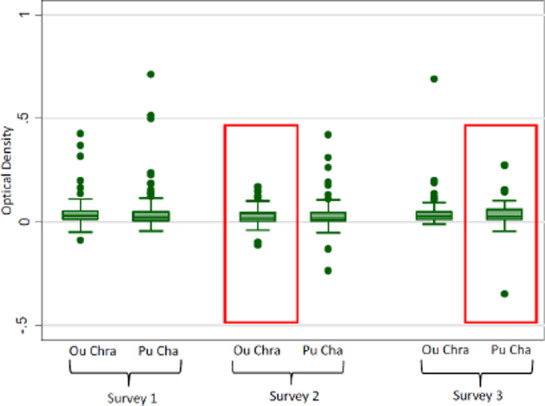
Box plots describing the range of corrected optical densities (OD minus the mean of two blank wells) of antibody responses to the gSG6 salivary protein. Red boxes denote the application of the intervention.

Figure 3 describes the all age logarithmic titres of *P. falciparum* and *P. vivax* AMA and MSP antibody responses by village and survey. The normalized ODs by village and survey showed minimal differences between either variable. There are slightly higher *P. falciparum* MSP119 ODs in Ou Chra than Pu Cha, otherwise very little difference was found. Figure 4 is a similar box plot but describes the corrected ODs for gSG6. Again, there was very little difference between age groups, villages or between surveys.

**Figure 5:**
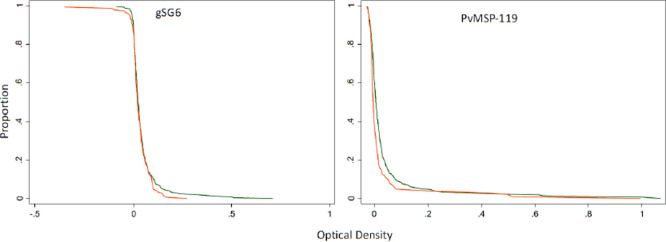
Reverse cumulative distribution plots of antibody responses to gSG6 salivary protein and PvMSP-119 at baseline (green line) and post intervention (red line). The graphs are constructed by plotting on the Y axis the proportion of subjects having an OD equal to or greater the level shown at each point along the x axis.

**Figure 6:**
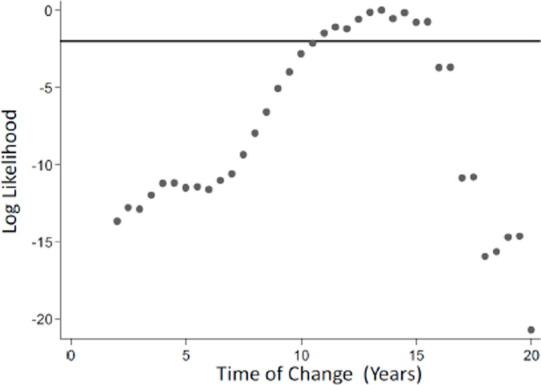
Profile likelihood plot (PLP) for both Pf antigens for Ou Chra and Pu Cha. PLP show the log likelihood of a catalytic conversion model allowing for a change in transmission occurring at iterative years. The maximum log likelihood is the time point at which a change in transmission is most likely to have occurred. The plot suggests a change in transmission occurred approximately 13 years ago.

Small drops in OD values from non-intervention to intervention villages were seen in the reverse cumulative distribution plots (RCDP). Figure 5 gives the reverse cumulative distribution plots of antibody responses to gSG6 salivary protein and *P. vivax* MSP-119 at baseline and after the intervention. Figure 6 shows the profile likelihood plot (PLP) for both *P. falciparum* antigens tested. The maximum log-likelihood is the time point at which a change in transmission is most likely to have occurred. It indicates that a change in transmission occurred approximately 13 years ago. The OD values for the intervention villages shifted to the left, in other words, the OD values were lower in the villages when the intervention was present, indicating a reduction in transmission. Among 1-5 year olds there was very little difference between baseline and intervention samples (*P. falciparum* MSP P=0.65, *P. falciparum* AMA P=0.53, *P. vivax* AMA P=0.86, *P. vivax* MSP P=0.73, gSG6 P=0.98). For all age groups combined, there was, however, a significant drop in *P. vivax* MSP119 ODs between baseline and intervention (*p*=0.000) and this was maintained when negative results were set to 0.001. Normalised optical densities for *P. falciparum* AMA1 also decreased between baseline and intervention but this was not significant (*p*=0.19) (*P. falciparum* MSP P=0.63, *P. vivax* AMA1 P=0.49, gSG6 P=0.77).

**Figure 7:**
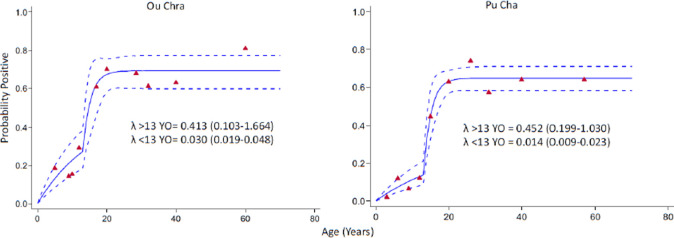
Anti-malaria seroconversion curve for antibodies to either PfAMA-1 or PfMSP-119 for participants in Ou Chra and Pu Cha across all surveys. A reversible catalytic model was fitted to age adjusted data using maximum likelihood to create seroconversion curves. Solid lines represent the fitted probability for being positive, dotted lines represent the 95% confidence interval for these fits, and triangles represent the observed proportion of seropositives per age decile. Seroconversion rates (SCR; λ) are presented on the graph. Change points were identified using profile likelihood plots. The plots identified a change point of 15 years for Ou Chra and 13 years for Pu Cha, models allowing for a change in SCR were significant (Likelihood ratio test: <0.05) compared to the model that did not allow a change.

A catalytic conversion model was used to fit combined antigen responses (i.e. positive to either MSP or AMA) with age-specific seroprevalence data and estimate seroconversion rates (SCR). Seroconversion curves showed the usual trend with increasing seroprevalence with age [33]. For *P. vivax* the relationship was almost linear. For both villages the fit between observed and expected values for *P. falciparum* improved when a predicted change in seroconversion 13 years previously was included in the model (Figure 7). Overall, however, prevalence and seroconversion rates were not affected by the intervention.

## 4 Discussion

The eight-fold difference in the number of *An. dirus* collected in the two villages probably reflects the proximity of the forest to the two villages. Indeed, the houses in Ou Chra with the highest densities of *An. dirus* were the houses at the edge of the village or close to the rice fields near the edge of the forest. The high numbers of *An. dirus* collected following the rain, and the absence of any indication of aestivation in the mosquito [17] indicates that transmission is likely to be greatest during the rainy season. In many places houses built off the ground have fewer anophelines in them [34]. The absence of a reduction in numbers collected as the height of the house increased and the anthropophilic tendency of the mosquito (as evidenced by its preponderance in light-traps inside houses but near absence in other collections) probably reflects its simian feeding tendencies in the forest.

The difference in numbers of *An. dirus* between the two villages, even before the introduction of the intervention, resulted in higher initial estimated prevalence rates in Ou Chra compared to Pu Cha although these were not quite significant (4/126 compared to 2/285 Fishers exact test p = 0.08). Indeed, apart from the difference in the reverse cumulative distribution plots of *P. vivax* no significant relationships were found in *Plasmodium* cases or seroconversion rates between villages, surveys or by intervention. Thus, both serological and parasitological data indicate that the intervention had little or no effect on parasite prevalence or transmission in the two villages but this might be an effect of low parasite rate and sample size. Adult males, who might spend more time unprotected in the forest at night, appeared to be at greater risk of getting *falciparum* malaria than women or young children.

The gSG6 assay is a relatively underdeveloped assay and may have lacked sensitivity. Thus, OD values for gSG6 showed very little difference between villages or age groups. The more pronounced increase in seroconversion rates with age may also be because of a higher incidence of the disease with age or it might, perhaps, be because the assay was originally optimised to work in African populations with high rates of *P. falciparum* transmission. It is also possible that with increasing exposure older people may tolerate the salivary gland proteins (since the antibody response against gSG6 would predominantly be from IgE). Such an effect would tend to mask any difference between age groups. The apparent change in transmission that occurred thirteen years before the survey is consistent with the arrival of many residents from non-endemic areas. Indeed, the residents in Ou Chra had generally been there for slightly longer than had residents in Pu Cha, which was also reflected in the seroconversion rates in the two villages.

The elimination of malaria from the greater Mekong region is a priority concern because of the possible spread of artemisinin-resistant parasites. Thus, even in low transmission areas, such as Ou Chra and Pu Cha, suitable and effective anti-vector measures, in addition to LLINs, are required. Enhanced information on the place where people acquire their parasites is also needed. The bias in *P. falciparum* cases among men over 17 years of age implies that they were being infected either before they went to bed or when they were in the forest. Without controlling transmission in the forest, either by using some novel technique, or by restricting access (and thereby reducing illegal deforestation) elimination in this region will be difficult.

## 5 Conclusion

There was no indication that the wide-scale application of metofluthrin dispensers to give complete coverage of all potential sitting places in all houses in either village affected malaria prevalence, despite reducing landing rates in outdoor collections elsewhere. The lack of an observed effect might, however, be due to the low rate of transmission in the study villages and to acquisition of the disease in forested areas away from the village. Studies in areas where transmission rates are higher would seem to be worthwhile.

## Supporting information

**Figure:**
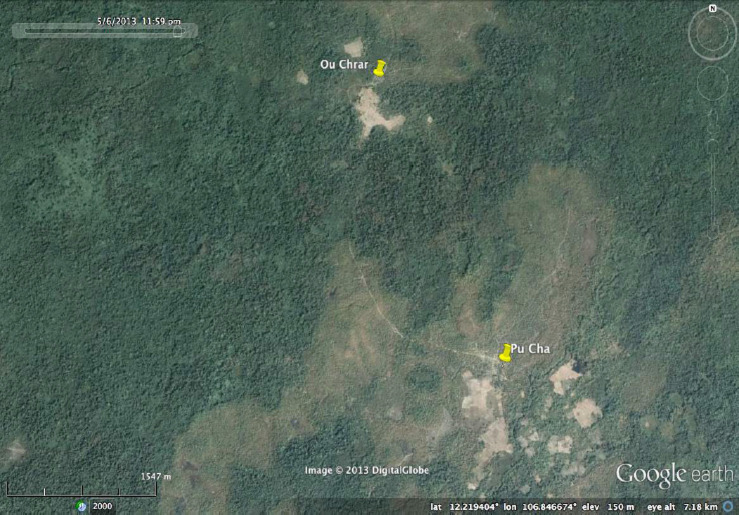
Location of Ou Chra and Pu Cha villages (Mondolkiri Province, Cambodia)

**Figure:**
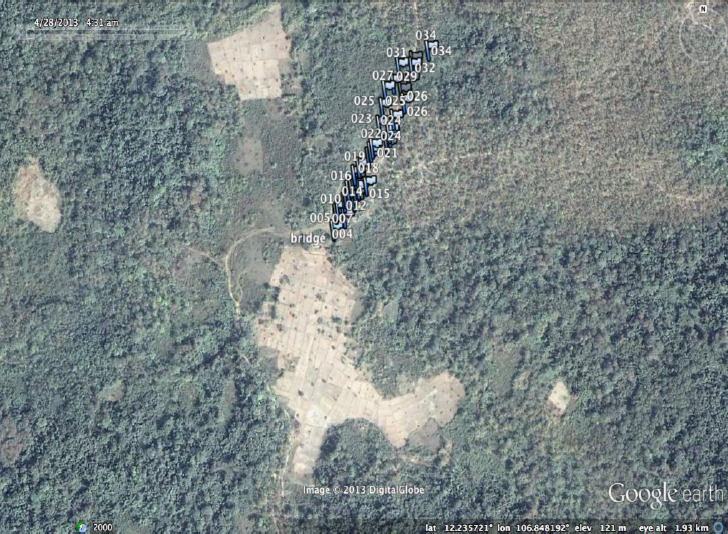
Ou Chra village with georeferenced houses

**Figure:**
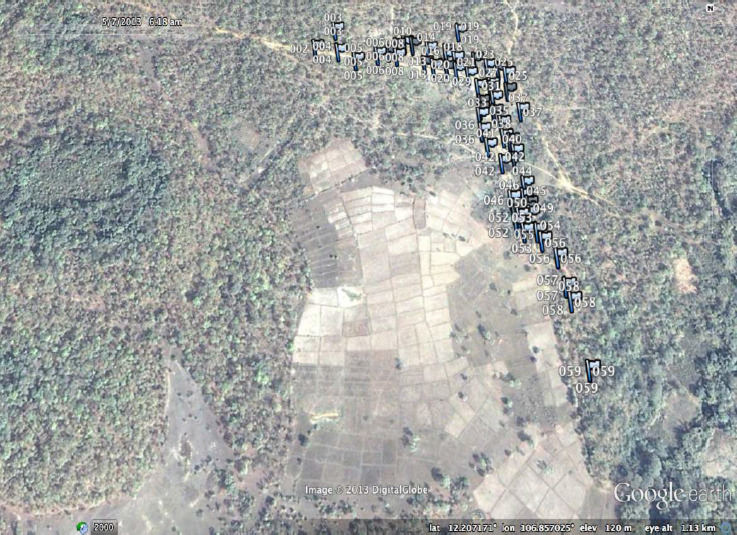
Pu Cha village with georeferenced houses
